# Roles of RNA m^6^A modifications in plant-virus interactions

**DOI:** 10.1007/s44154-023-00133-x

**Published:** 2023-12-18

**Authors:** Hao He, Mingxuan Jia, Jie Liu, Xueping Zhou, Fangfang Li

**Affiliations:** 1 State Key Laboratory for Biology of Plant Diseases and Insect Pests, Institute of Plant Protection, Chinese Academy of Agricultural Sciences, Beijing, China; 2 State Key Laboratory of Rice Biology, Institute of Biotechnology, Zhejiang University, Hangzhou, Zhejiang China

**Keywords:** Plant virus, N^6^-methyladenosine (m^6^A) RNA modification, Plant-virus interactions

## Abstract

Viral RNAs have been known to contain N^6^-methyladenosine (m^6^A) modifications since the 1970s. The function of these modifications remained unknown until the development of genome-wide methods to map m^6^A residues. Increasing evidence has recently revealed a strong association between m^6^A modifications and plant viral infection. This highlight introduces advances in the roles of RNA m^6^A modifications in plant-virus interactions.

## Main text

**Fig. 1:**
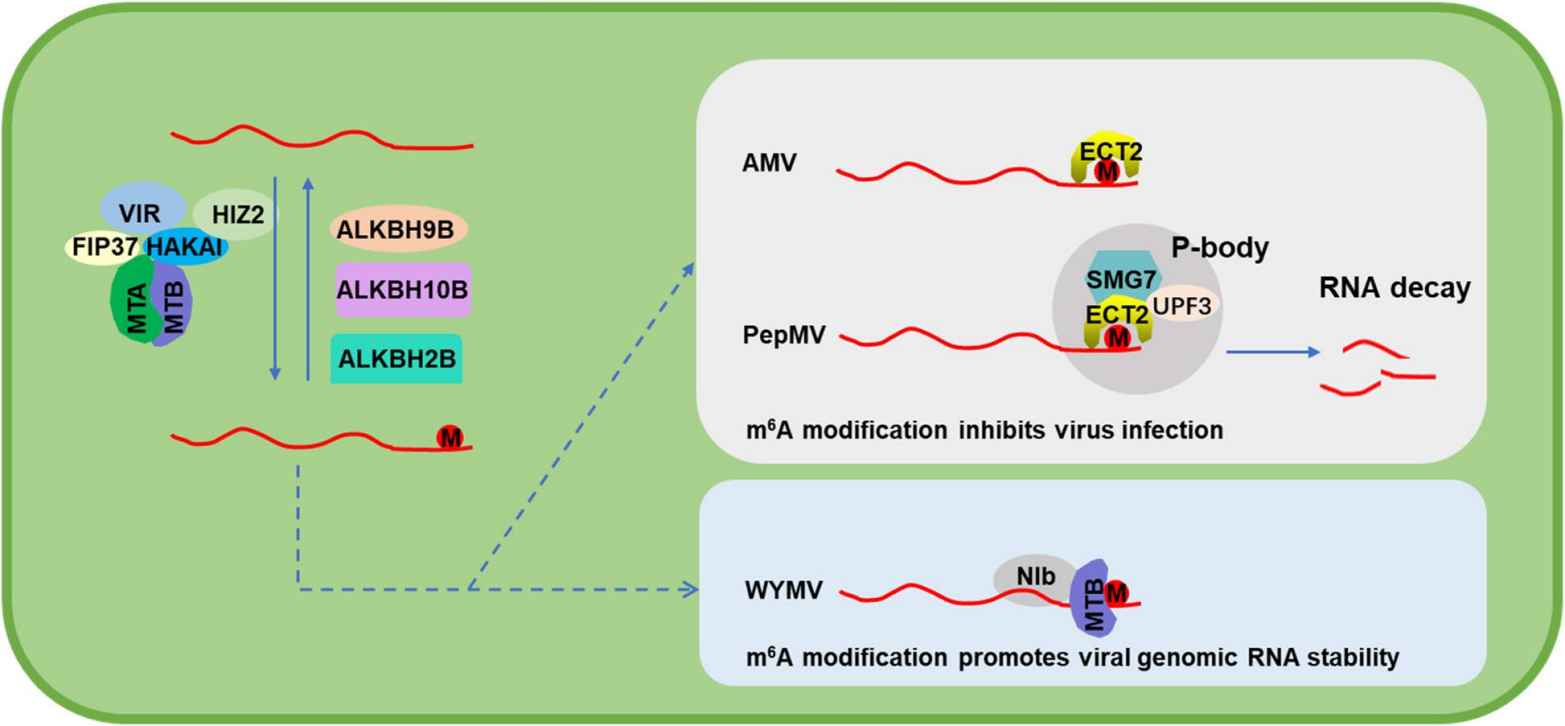
Roles of m^6^A modifications in plant virus infection. m^6^A mRNA modification is catalyzed by a conserved m^6^A methyltransferase complex in plants containing MTA, MTB, FIP37, VIR, HAKAI, and HIZ2. The interactions among m^6^A components are supported by recent studies. m^6^A is removed by m^6^A demethylase, which belongs to the AlkB family and is recognized by ECT (evolutionarily conserved C-terminal regions) proteins in plants. Plant viral RNA undergoes m^6^A modification during viral infection. The addition of m^6^A in plant viral mRNAs has different functions in distinct viral life cycles. In some cases, m^6^A is shown to serve as a negative regulator in viral infection. For example, the m^6^A demethylase AtALKBH9B in Arabidopsis was found to interact with the envelope protein of alfalfa mosaic virus (AMV) and promote systemic viral invasion. Moreover, the *ECT2/ECT3/ECT4/ECT5* module in Arabidopsis reduces AMV resistance, and the increased AMV resistance of *alkbh9b* mutants can be reverted by mutation of *ECT2/ECT3/ECT5*. The m^6^A modifications on PepMV genomic RNA were also found in infected *Nicotiana benthamiana* and *Solanum lycopersium*. The m^6^A writers MTA, HAKAI, and m^6^A readers NbECT2A/B/C negatively regulate pepino mosaic virus (PepMV) infection. NbECT2A/2B/2C can further mediate the PepMV RNA degradation in the processing body by recruiting RNA-decay-related host factors. However, some viruses acquire m^6^A modifications in viral RNA to promote viral genomic RNA stability and infection. For example, *Triticum aestivum* m^6^A methyltransferase B (TaMTB), a positive regulator for WYMV infection, interacts with wheat yellow mosaic virus (WYMV) NIb to stabilize the viral RNA. MTA, mRNA adenosine methylase A; MTB, mRNA adenosine methylase A; FIP37, FKBP12 Interacting Protein 37; VIR, VIRILIZER; HIZ2, HAKAI-interacting zinc finger protein 2; P-body, processing body; UPF3, up-frameshift protein 3; SMG7, suppressor with morphogenetic effects on genitalia 7

N^6^-methyladenosine (m^6^A) is the most pivotal internal modification and is widely present in mRNA, rRNAs, and long non-coding RNA (lncRNA) in eukaryotes (Boccaletto et al. 2022). The modification has shown to be reversible and is catalyzed by methyltransferases (writers), removed by demethylases (erasers), and recognized by m^6^A binding proteins (readers) (Fu et al. 2014). m^6^A has been demonstrated to play a vital role in viral infection in mammals. In some cases, m^6^A is shown to serve as a negative regulator in viral infection (Gokhale et al. 2016; Lichinchi et al. 2016b). Nevertheless, some viruses can also take advantage of this modification for viral enhancement (Kennedy et al. 2016; Lichinchi et al. 2016a), indicating the pivotal role of m^6^A modification in host-virus interactions. In the meantime, mounting evidence shows that m^6^A modification also occurs in plant viruses, and its roles in the arms race of plants and viruses have been uncovered in recent work (Fig. 1).

As early as the 1970s, m^6^A modification was identified in viral RNAs, such as influenza virus (Krug et al. 1976). In the last ten years, with the development of multiple detection technologies, the presence of m^6^A was reported in the genomic RNA of several plant viruses. Two members of the *Bromoviridae* family, alfalfa mosaic virus (AMV) and cucumber mosaic virus (CMV) have been reported to contain m^6^A modifications in the genomic RNAs by the methylated RNA immunoprecipitation sequencing (MeRIP-Seq) (Martínez-Pérez et al. 2017). Furthermore, two of these putative m^6^A-sites in the 3’-UTR of AMV RNA3 were reported to be involved in viral replication/accumulation and in vivo plus-strand accumulation (Alvarado-Marchena et al. 2022). The m^6^A distribution patterns on viral genomic RNA of rice black streaked dwarf virus (RBSDV) and rice stripe virus (RSV) were also revealed by Zhang and his colleagues. Clustered m^6^A peaks in the 5′ terminal of RBSDV genomic S1, S2, S3, S4, S5, S6, S9, and S10 and some discrete peaks in RSV RNA1 to RNA4 were observed (Zhang et al. 2021a). Two and four m^6^A peaks were significantly enriched in plum pox virus (PPV) and potato virus Y (PVY) genomes by MeRIP-seq (Yue et al. 2022). Four obvious m^6^A peaks in the coding region of RNA1 and one m^6^A peak in the 3’ terminal of RNA2 were found in the genomic RNAs of wheat yellow mosaic virus (WYMV). m^6^A modification occurring on the 6800th A in the WYMV RNA1 was further identified to be involved in the stability of viral *CP* transcripts (Zhang et al. 2022). The m^6^A modifications of pepino mosaic virus (PepMV) genomic RNA in infected *Nicotiana benthamiana* and *Solanum lycopersium* leaves were also mapped in the viral 3’-terminal in the latest study (He et al. 2023a).

Viral infection has been known to affect host m^6^A dynamics in mammals (Gokhale et al. 2016; Lichinchi et al. 2016a). Studies of the m^6^A dynamics in plant-virus interactions have also been revealed in the last three years. With an ultra-high performance liquid chromatography coupled with high-resolution tandem mass spectrometry (UHPLC − HR − MS/MS) method, Li et al. found that levels of m^6^A in *Nicotiana tabacum* appear to be decreased by tobacco mosaic virus (TMV) infection, which was in correspondence with the increased mRNA expression of the putative demethylase and decreased putative methyltransferase level after TMV infection (Li et al. 2018). In agreement with this finding, *N. benthamiana* m^6^A levels were reduced by infection of PPV and PVY (Yue et al. 2022). On the contrary, Zhang et al. analyzed the high-quality m^6^A methylomes in rice plants infected with RSV and RBSDV. They found that the m^6^A modification levels of rice mRNAs were enriched under infection of these two viruses (Zhang et al. 2021a). Interestingly, the m^6^A levels significantly increased to 1.397-fold in susceptible watermelon plants 24 h after cucumber green mottle mosaic virus (CGMMV) infection but significantly decreased to 0.757-fold at 48 h in resistant watermelons (He et al. 2021). These studies indicate that host m^6^A levels can be altered by viral infection, which might further affect the gene expression of hosts. CGMMV infection regulated the expression of 59 host cell genes by affecting the deposition of m^6^A, which involved multiple roles and signaling pathways such as resistance response, secondary biosynthesis and metabolism, and RNA processes. The high-quality m^6^A methylomes in rice plants infected with RSV and RBSDV were also analyzed, and several antiviral pathway-related genes, such as RNA silencing-, resistance-, and fundamental antiviral phytohormone metabolic-related genes, were m^6^A methylated upon RSV and RBSDV infection (Zhang et al. 2021a). In addition, transcriptome-wide m^6^A profiling in WYMV-infected resistant wheat variety and WYMV-infected sensitive wheat variety revealed significant changes in m^6^A and mRNA levels associated with plant defense responses (Zhang et al. 2021b). These studies deepen our understanding of the significant role of m^6^A in altering hosts’ physiological and pathological status in the context of viral infection.

In some cases, adding m^6^A in plant viral RNAs has antiviral function in distinct viral life cycles. Suppression of AtALKBH9B increased the relative abundance of m^6^A in the AMV genome, impairing the systemic invasion of the plant (Martínez-Pérez et al. 2017). Consistent with the above result, the downregulation of *N. benthamiana* AlkB homologs of the plant-specific ALKBH9 clade caused a significant decrease in PPV and PVY accumulation (Yue et al. 2022). Furthermore, overexpression of NbMETTL homologs (NbMETTL1 and NbMETTL2) promoted PPV resistance in *N. benthamiana* (Yue et al. 2023). Similarly, after *LsMETTL3* and *LsMETTL14*, which encode m^6^A RNA methyltransferase in small brown planthopper (SBPHs), were knocked down, the titer of RBSDV in the midgut cells of SBPHs increased significantly (Tian et al. 2021).

Although m^6^A methylation plays an anti-viral role in plant viral infection, the underlying molecular mechanisms still need further study to reconcile these differing observations. Notably, the primary mechanism by which m^6^A exerts its effects is determined by which m^6^A-binding proteins (m^6^A readers) are recruited (Meyer and Jaffrey 2017). Recently, Martínez-Pérez et al. found that mutation of the *ECT2/ECT3/ECT4/ECT5* module in Arabidopsis reduced AMV resistance and that the increased AMV resistance of *alkbh9b* mutants could be reverted by deficiencies of *ECT2/ECT3/ECT5*, indicating that the m^6^A-reader axis constituted a novel basal antiviral defense layer in plants (Martínez-Pérez et al. 2023). Supporting this conclusion, He et al. also found that the cytoplasmic YTH-domain family proteins NbECT2A/2B/2C could mediate the PepMV RNA degradation in the processing body by recruiting RNA-decay related host factors, including SMG7 and UPF3 proteins, thereby inhibiting virus infection through the RNA decay-related machinery (He et al. 2023a).

However, some viruses have also evolved anti-defense strategies to counterattack the plant defense responses mediated by m^6^A modification. For example, the PepMV-encoded RNA-dependent RNA polymerase (RdRP) exploits the autophagy pathway by interacting with an autophagy core protein, SlBeclin1, to promote the autophagic degradation of the SlHAKAI protein, thereby inhibiting the m^6^A modifications-mediated plant defense responses (He et al. 2023b). In addition, some viruses might acquire m^6^A modifications in viral RNA as a unique mechanism to promote viral genomic RNA stability and infection. A recently characterized susceptibility gene encoding *Triticum aestivum* m^6^A methyltransferase B (TaMTB) is identified as a positive regulator for WYMV infection. TaMTB is localized in the nucleus and is translocated into the cytoplasmic viral replication complexes by interacting with WYMV NIb to upregulate the m^6^A level of WYMV RNA1 and stabilize the viral RNA, thus promoting viral infection (Zhang et al. 2022). Interestingly, several plant viruses have been found to contain AlkB protein homologs or domains belonging to m^6^A demethylases, indicating that these viruses may exploit this as a novel counter-defense mechanism (Yue et al. 2022).

The work above demonstrates that plant RNA viruses undergo m^6^A modification during viral infection. Despite much progress, most studies to date focus on the qualitative and quantitative analyses of m^6^A using mass spectrometry (MS) or MeRIP-seq, which cannot enable absolute quantification of m^6^A at single-base resolution. Therefore, developing new techniques to map m^6^A modification with single-base resolution will help further dissect the roles of m^6^A modification in plant-virus interactions. Considering that the knockout of most m^6^A methyltransferases resulted in embryonic death, using small molecule inhibitors of m^6^A methyltransferases might help study the m^6^A modification in plant and virus interactions. In most cases, m^6^A modification plays an antiviral role in plant viral infection. However, the specific mechanisms still need further investigation. Of note, m^6^A is closely related to the alteration of hosts’ physiological and pathological status during plant viral infection. A comprehensive understanding of m^6^A methylation in plant-virus interactions and the crosstalk between m^6^A modification and other immunity-related pathways must be further explored. In addition, further studies will be necessary to answer whether m^6^A methylation occurs in the mRNA of plant DNA viruses.

## Data Availability

All the data supporting the claims contained in this manuscript are provided in the submission and can be shared publicly after acceptance of the manuscript for publication by Stress Biology.

## Abbreviations

m^6^A: N^6^-methyladenosine
lncRNA: Long non-coding RNA
AMV: Alfalfa mosaic virus
CMV: Cucumber mosaic virus
MeRIP-Seq: Methylated RNA immunoprecipitation sequencing
RBSDV: Rice black streaked dwarf virus
RSV: Rice stripe virus
PPV: Plum pox virus
PVY: Potato virus Y
WYMV: Wheat yellow mosaic virus
CP: Coat protein
PepMV: Pepino mosaic virus
UHPLC-HR-MS/MS: Ultra-high performance liquid chromatography coupled with high-resolution tandem mass spectrometry
TMV: Tobacco mosaic virus
CGMMV: Cucumber green mottle mosaic virus
SBPH: Small brown planthopper
RdRP: RNA-dependent RNA polymerase
TaMTB: *Triticum aestivum* M^6^A methyltransferase B
ECT: Evolutionarily conserved C-terminal regions
MS: Mass spectrometry

## References

[CR1] Alvarado-Marchena L, Martínez-Pérez M, Úbeda JR, Pallas V, Aparicio F (2022). Impact of the potential m^6^A modification sites at the 3'UTR of alfalfa mosaic virus RNA3 in the viral infection. Viruses.

[CR2] Boccaletto P, Stefaniak F, Ray A, Cappannini A, Mukherjee S, Purta E, Kurkowska M, Shirvanizadeh N, Destefanis E, Groza P, Avşar G, Romitelli A, Pir P, Dassi E, Conticello SG, Aguilo F, Bujnicki JM (2022). MODOMICS: a database of RNA modification pathways. 2021 update. Nucleic Acids Res.

[CR3] Fu Y, Dominissini D, Rechavi G, He C (2014). Gene expression regulation mediated through reversible m^6^A RNA methylation. Nat Rev Genet.

[CR4] Gokhale NS, McIntyre ABR, McFadden MJ, Roder AE, Kennedy EM, Gandara JA, Hopcraft SE, Quicke KM, Vazquez C, Willer J, Ilkayeva OR, Law BA, Holley CL, Garcia-Blanco MA, Evans MJ, Suthar MS, Bradrick SS, Mason CE, Horner SM (2016). N^6^-methyladenosine in *Flaviviridae* viral RNA genomes regulates infection. Cell Host Microbe.

[CR5] He H, Ge L, Chen Y, Zhao S, Li Z, Zhou X, Li F (2023). m^6^A modification of plant virus enables host recognition by NMD factors in plants. Sci China Life Sci.

[CR6] He H, Ge L, Li Z, Zhou X, Li F (2023). Pepino mosaic virus antagonizes plant m^6^A modification by promoting the autophagic degradation of the m^6^A writer HAKAI. BaBIOTECH.

[CR7] He Y, Li L, Yao Y, Li Y, Zhang H, Fan M (2021). Transcriptome-wide N^6^-methyladenosine (m^6^A) methylation in watermelon under CGMMV infection. BMC Plant Biol.

[CR8] Kennedy EM, Bogerd HP, Kornepati AVR, Kang D, Ghoshal D, Marshall JB, Poling BC, Tsai K, Gokhale NS, Horner SM, CμLlen BR (2016). Posttranscriptional m^6^A editing of HIV-1 mRNAs enhances viral gene expression. Cell Host Microbe.

[CR9] Krug RM, Morgan MA, Shatkin AJ (1976). Influenza viral mRNA contains internal N^6^-methyladenosine and 5’-terminal methylguanosine in cap structures. J Virol.

[CR10] Li Z, Shi J, Yu L, Zhao X, Ran L, Hu D, Song B (2018). N^6^-methyl-adenosine level in *Nicotiana tabacum* is associated with tobacco mosaic virus. J Virol.

[CR11] Lichinchi G, Gao S, Saletore Y, Gonzalez GM, Bansal V, Wang Y, Mason CE, Rana TM (2016). Dynamics of the human and viral m^6^A RNA methylomes during HIV-1 infection of T cells. Nat Microbiol.

[CR12] Lichinchi G, Zhao BS, Wu Y, Lu Z, Qin Y, He C, Rana TM (2016). Dynamics of human and viral RNA methylation during zika virus infection. Cell Host Microbe.

[CR13] Martínez-Pérez M, Aparicio F, Arribas-Hernández L, Tankmar MD, Rennie S, von Bülow S, Lindorff-Larsen K, Brodersen P, Pallas V (2023). Plant YTHDF proteins are direct effectors of antiviral immunity against an N^6^-methyladenosine-containing RNA virus. EMBO J.

[CR14] Martínez-Pérez M, Aparicio F, López-Gresa MP, Bellés JM, Sánchez-Navarro JA, Pallás V (2017). Arabidopsis m^6^A demethylase activity modulates viral infection of a plant virus and the m^6^A abundance in its genomic RNAs. Proc Natl Acad Sci USA.

[CR15] Meyer KD, Jaffrey SR (2017). Rethinking m^6^A readers, writers, and erasers. Annu Rev Cell Dev Biol.

[CR16] Tian S, Wu N, Zhang L, Wang X (2021). RNA N^6^-methyladenosine modification suppresses replication of rice black streaked dwarf virus and is associated with virus persistence in its insect vector. Mol Plant Pathol.

[CR17] Yue J, Lu Y, Sun Z, Guo Y, San León D, Pasin F, Zhao M (2023). Methyltransferase-like (METTL) homologues participate in Nicotiana benthamiana antiviral responses. Plant Signal Behav.

[CR18] Yue J, Wei Y, Sun Z, Chen Y, Wei X, Wang H, Pasin F, Zhao M (2022). AlkB RNA demethylase homologues and N^6^-methyladenosine are involved in Potyvirus infection. Mol Plant Pathol.

[CR19] Zhang K, Zhuang X, Dong Z, Xu K, Chen X, Liu F, He Z (2021). The dynamics of N^6^-methyladenine RNA modification in interactions between rice and plant viruses. Genome Biol.

[CR20] Zhang T, Shi C, Hu H, Zhang Z, Wang Z, Chen Z, Feng H, Liu P, Guo J, Lu Q, Zhong K, Chen Z, Liu J, Yu J, Chen J, Chen F, Yang J (2022). N^6^-methyladenosine RNA modification promotes viral genomic RNA stability and infection. Nat Commun.

[CR21] Zhang T, Wang Z, Hu H, Chen Z, Liu P, Gao S, Zhang F, He L, Jin P, Xu M, Chen J, Yang J (2021). Transcriptome-wide N^6^-methyladenosine (m^6^A) profiling of susceptible and resistant wheat varieties reveals the involvement of variety-specific m^6^A modification involved in virus-host interaction pathways. Front Microbiol.

